# Data-Driven Modeling and the Influence of Objective Function Selection on Model Performance in Limited Data Regions

**DOI:** 10.3390/ijerph17114132

**Published:** 2020-06-10

**Authors:** Thelma Dede Baddoo, Zhijia Li, Yiqing Guan, Kenneth Rodolphe Chabi Boni, Isaac Kwesi Nooni

**Affiliations:** 1 State Key Laboratory of Hydrology-Water Resources and Hydraulic Engineering, College of Hydrology and Water Resources, Hohai University, Nanjing 210098, China; 2 College of Hydrology and Water Resources, Hohai University, Nanjing 210098, China; zjli@hhu.edu.cn (Z.L.); yiqingguan@hhu.edu.cn (Y.G.); 3 College of Computer and Information Engineering, Hohai University, Nanjing 211100, China; boni_kenneth@yahoo.fr; 4 School of Geographical Sciences, Nanjing University of Information Science & Technology, Nanjing 210044, China; nooni25593@alumni.itc.nl; 5 Binjiang College, Nanjing University of Information Science & Technology, No.333 Xishan Road, Wuxi 214105, China

**Keywords:** data-driven modeling, objective function selection, Zhidan watershed, IHACRES, hydromad, China

## Abstract

The identification of unit hydrographs and component flows from rainfall, evapotranspiration and streamflow data (IHACRES) model has been proven to be an efficient yet basic model to simulate rainfall–runoff processes due to the difficulty in obtaining the comprehensive data required by physical models, especially in data-scarce, semi-arid regions. The success of a calibration process is tremendously dependent on the objective function chosen. However, objective functions have been applied largely in over daily and monthly scales and seldom over sub-daily scales. This study, therefore, implements the IHACRES model using ‘hydromad’ in R to simulate flood events with data limitations in Zhidan, a semi-arid catchment in China. We apply objective function constraints by time aggregating the commonly used Nash–Sutcliffe efficiency into daily and hourly scales to investigate the influence of objective function constraints on the model performance and the general capability of the IHACRES model to simulate flood events in the study watershed. The results of the study demonstrated the advantage of the finer time-scaled hourly objective function over its daily counterpart in simulating runoff for the selected flood events. The results also indicated that the IHACRES model performed extremely well in the Zhidan watershed, presenting the feasibility of the use of the IHACRES model to simulate flood events in data scarce, semi-arid regions.

## 1. Introduction

Arid and semi-arid regions are recurrently prone to be dominated by extreme rainfall events with a high degree of spatial variability, usually resulting in rapid response profiles [1]. Thus, accurate modeling of these events would facilitate proper flood forecasting in these areas to save lives and property. Flood modeling remains one of the most challenging and important tasks of operational hydrology due to spatial and temporal variations in rainfall distribution and the tremendously complex and highly nonlinear nature of the rainfall–runoff relationship [2]. The ability to model river flow quickly and precisely is of critical importance in flood modeling [3], and the need to better understand the earth’s hydrological system has led to the rise of numerous conceptual and physically based models.

Physically based models assume that the nature of the physical factors affecting streamflow variations in a catchment are known or that some realistic approximations of the underlying physics can be generated, and thus require detailed knowledge of watershed characteristics, information that commonly is not known a priori [4]. This makes the application and calibration of conceptual and physically based models associated with several difficulties: requiring sophisticated mathematical tools, considerable amounts of calibration data and some degree of expertise and experience with the model [5,6,7]. They are also characterized by often possessing a significant number of parameters which could be challenging to measure or estimate due to insufficient field data [8,9].

The fact that the comprehensive hydrological data required by conceptual or physically based models are rarely available in most watersheds has led to the rapid development of data-driven models (DDMs) in hydrology and environmental sciences. Various developments in the recent data-driven computing models have revealed their immense modeling capabilities amidst limited and flawed input space [10]. This is because they are based on analyzing the data about a system without explicit knowledge of the physical behavior by using machine-learning algorithms to determine the relationship between a system’s inputs and outputs [11,12,13]. Therefore, DDMs require the least amount of different kinds of data for model calibration, are often cheaper and simpler to implement and many times with fewer parameters than their physically based counterparts [14,15].

The identification of unit hydrographs and component flows from rainfall, evapotranspiration and streamflow data (IHACRES) model, a parsimonious hybrid conceptual-data-driven rainfall–runoff model [16], developed by the Institute of Hydrology and the Australian National University’s Center for Resource and Environmental Studies [17] has been proven to be an efficient yet basic rainfall–runoff model to better simulate rainfall–runoff processes. It uses the simplicity of the metric model, requiring typically between 5 and 8 parameters, to reduce the parameter uncertainty inherent in hydrological models and has a high predictive accuracy, making it easier than complex models to be applied in poorly gauged, arid and semi-arid regions [1,16,18,19,20].

However, the model has seldom been used in modeling flood events, with these previous studies mostly focused on the performance of the model using continuous daily or monthly rainfall and runoff data especially in semi-arid regions (see Croke and Jakeman [1] and Ye et al. [21]). Studies performed using the IHACRES in event simulations are either not representative enough to ascertain the versatility and robustness of the model (for example, Abushandi and Merkel [22]) or resulted in unsatisfactory model results (work by Kan et al. [23]).

The time step dependency of parameters in hydrological modeling has been studied over time. These studies have often highlighted the performance of models using parameters derived from the one-time step for simulating runoff at another time step (mostly from daily or monthly time steps to sub-daily time steps (for example, Littlewood and Croke [24], Reynolds et al. [25], Bennett et al. [26], Littlewood et al. [27] and Littlewood and Croke [28]). The modeling in these studies, however, is done employing a somewhat long calibration data time series to obtain parameters. Then the temporal data resolutions are up-scaled or downscaled to compare parameter stability and independence, mostly on a continuous modeling basis and, therefore, cases where sub-daily event data are available, but in limited supply have not been researched.

The assessment of hydrologic model behavior and performance is generally made and reported through comparisons of simulated and observed variables, which in the case of hydrology are comparisons made between simulated and measured streamflow at the catchment outlet [29], referred to as the calibration process. Objective functions or ‘goodness of fit’ criteria, which are mathematical measures, are used to provide an objective assessment of the “closeness” of the simulated behavior to the observed measurements [29]. Servat and Dezzetter [30] define an objective function as a reference numerical “quantity” enabling calibration to be improved. Objective functions are used in the generation of the response surface or objective criteria, which in many cases are geared towards minimizing or maximizing the selected objected function to measure the performance of the model calibration [31,32]. In general, the purpose of the objective function in an optimization procedure is to generate parameters in the response surface to obtain the largest or least objective function value depending on its acceptable optimal ranges. Research has shown that the success of a calibration process is tremendously dependent on the objective function chosen as a calibration criterion [33,34]. That model performance is more closely related to the model structure and the objective function used than model complexity or calibration data length [31]. It has also been indicated that simulated hydrographs can be quite different, depending on which criterion is selected as the objective function [35].

The Nash–Sutcliffe Efficiency (NSE or E or R^2^) [36] is a very commonly used objective function and hydrological model performance assessment measure. It is recommended for use in literature by ASCE [37], Legates and McCabe [38] and Moriasi et al. [39] and suggested as the best objective function for reflecting the overall fit of a hydrograph by Servat and Dezzetter [30]. The application of the Nash–Sutcliffe efficiency (NSE) measure as the objective function in any optimization process is to iteratively modify the model parameters in the parameter response space to obtain the highest NSE value to improve the model simulation.

Legates and McCabe [38] stated, however, that NSE is overly sensitive to extreme values because the differences between the observed and predicted values are calculated as squared values. Therefore, larger values in a time series are strongly overestimated, whereas lower values are neglected. An example in flood event modeling is when there is some challenge of a model to simulate peak flow (one extreme outlier) and the NSE obtained is unsatisfactory due to the consideration of the extreme low or high simulated peak flow and the rest of the accurately simulated low flow periods at the start and end of the event are neglected by the NSE measure. Or in contrast, where the NSE estimated is satisfactory due to the accurate simulation in peak flow and the poor low flow simulations at the start and end of the event are neglected.

In addition. in much literature, NSE has been estimated mostly on daily and monthly scales (literature review by Moriasi et al. [39]), where the NSE is estimated over the entire time–space. Viney et al. [40] also state that modelers mostly calibrate their models using an objective function at the daily or monthly time step. This means that the temporal structure of the NSE objective function is applied to be maximized over daily or monthly scales over the entire calibration period. In such cases, using NSE at a monthly scale as calibration criteria on one (1) year of data of daily time step, estimates the NSE over a 12-month time scale, neglecting the three hundred and sixty-five (365) days built into the data. This condition, in cases of modeling with limited data, especially in data-scarce regions, may result in poor model performance because of the neglect of the intrinsically fine temporal resolution in the data. In such cases, a refined form of the objective function is essential; that is, the NSE estimated at a much finer scale is recommended to ameliorate this oversensitivity.

Hydrological modeling studies have generally revealed poor model performance results in the Zhidan catchment, a semi-arid catchment located in Shaanxi Province in northern China, as observed by Miao et al. [41], Liu et al. [42] and Li et al. [43]. However, most of the studies have involved the application of distributed or semi-distributed physical models where better model performance means the requirement of additional calibration data, which could be unavailable.

To the best of our knowledge, studies involving time aggregation of objective functions to obtain improved model performance when the commonly used model simulation processes fail due to data limitations are lacking. This study, therefore, implements the IHACRES model to simulate a series of flood events over some years representing changes in land-use and landcover settings in the Zhidan catchment using the Nash Sutcliffe Efficiency as the objective function of the optimization procedure for parameter generation and fitting. The study applies objective function constraints on the Nash efficiency objective function (R^2^ or rsq in this study) by time aggregating it into daily (rsq_daily) and hourly (rsq_hourly) objective functions during the modeling process to investigate (1) the influence of objective function constraints on the model performance and (2) the general capability of the IHACRES model to simulate flood events in a data-scarce semi-arid region.

This study focuses on limited data and a data-driven model because of data availability gaps in many parts of the world, especially in semi-arid regions where data are considerably scarce, and yet there is the need to properly understand watershed processes in light of climate change impacts, vulnerability and adaptation.

## 2. Materials and Methods

### 2.1. Study Area

**Figure 1:**
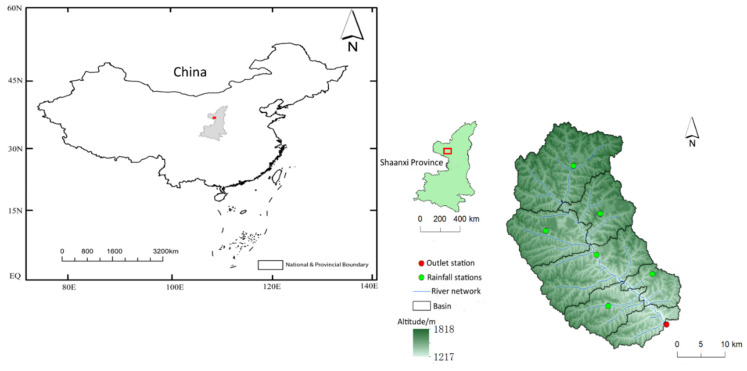
Map of China showing the location of the study area and the Zhidan watershed area showing the river network with the meteorological stations and the hydrometeorological station (outlet).

The study was conducted in the Zhidan watershed, geographically located in Shaanxi Province, China (on latitude 36°49′ N, longitude 108°46′ E) [44]. The watershed (a subset of the Yellow River watershed) is about 774 km^2^ [41] and includes a main river length of 81.3 km with an average elevation of approximately 1230 m [44]. The area is a mountainous catchment with scant vegetation; the soil texture of the catchment consists of loess soil, silt soil and saline soil. As a result, it suffers from severe soil erosion [23,44]. The area lies in the middle temperate arid region and experiences a continental monsoon climate classification according to the Köppen–Geiger climate and Kan et al. [23]. The watershed has six meteorological stations and one hydrometeorological station (outlet) located downstream of the river (Figure 1).

### 2.2. Data

Data on yearly flood events that occurred in the study watershed for the period of 2000–2010 in the form of hourly average areal rainfall and hourly point streamflow data at the catchment outlet for each flood event of each year were obtained from the local hydrological bureau of the Zhidan watershed. In addition, daily evapotranspiration data in the watershed from 2000–2010 were obtained and transformed into hourly evapotranspiration by simple disaggregation (by dividing the daily values by 24, as done by Bennett et al. [26] with rainfall data) for use in this study. This was done because hourly evapotranspiration data were unavailable during this study, and the identification of unit hydrographs and component flows from rainfall, evapotranspiration and streamflow data (IHACRES) model requires temperature or evapotranspiration data for model calibration. The Zhidan watershed is considered data-scarce because continuous time series data in the watershed over a long period (data such as those before the 2000s and after 2010) are unavailable. For example, hourly data (rainfall and runoff only) were available for only the flood events for the period of 2000–2010 without hourly data on evapotranspiration for the flood events. In addition, the continuous-time series of daily data available (which is available from 2000 to 2010) is mostly incomplete with missing data. Furthermore, another consideration of data limitation in the Zhidan watershed is the short duration of the flash floods, which occur in the watershed due to its semi-arid climate. These flood events have only a few data points that pose challenges to models to adapt to the quick runoff response in the watershed.

Six flood events with limited data (5 events with less than 72 h of data and 1 event with less than 24 h of data) spanning some years showing different landcover changes in the watershed were selected. This was implemented to clarify the reason for the methodology applied in this study (that is, the need for time aggregation of objective functions in modeling processes) and to investigate the flexibility and robustness of the IHACRES model to simulate flood events.

### 2.3. Methods

Exploratory data analyses (EDA) in this study in the form of tables and graphs were performed on the data to have a first view of the rainfall–runoff behavior in the study watershed in terms of the rainfall and runoff characteristics to assess the changes taking place in the catchment better and to understand the flood processes of the Zhidan study watershed.

The IHACRES model comprises two modules in series (a nonlinear loss module and a linear routing module): the first module operates nonlinearly to calculate effective rainfall (that portion which eventually reaches the stream prediction point) from total rainfall and evapotranspiration or temperature data as a surrogate (nonlinear loss module) [1,45]. Various versions of the IHACRES nonlinear loss module have been developed by Ye et al. [21] for ephemeral streams, Post and Jakeman [46] for assessing different catchment attributes and hydrological response relationships and Evans and Jakeman [47] first introduced the catchment moisture deficit (CMD) version which was revised by Croke and Jakeman [16].

**Figure 2:**
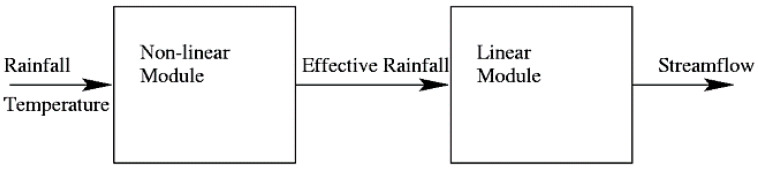
Generic structure of the identification of unit hydrographs and component flows from rainfall, evapotranspiration and streamflow data (IHACRES) model. Retrieved from Croke and Jakeman [1].

The remaining module of the IHACRES model operates linearly to convert and route effective rainfall to total streamflow, having partitioned it into quick and slow flow components through any configuration of stores in parallel or series [1,45]. The configuration of stores is identified from the time series of rainfall and discharge. The configuration of stores is typically either one store only, representing ephemeral streams or two in parallel, for catchments with significant baseflow components, allowing baseflow or slow-flow to be represented as well as quick flow [1]. Only rarely does a more complex configuration than this improve the fit to discharge measurements [48]. The generic structure of the IHACRES model is presented in Figure 2.

The IHACRES model has six (6) parameters used in runoff simulation with an additional two (2) parameters for ephemeral streams [20,46]: the catchment drying rate at a reference temperature, $\tau_{w}$, which is a time constant for the decline in the catchment wetness index, the temperature modulation parameter, f, which regulates the degree of evaporation dependence of the loss time constant, the mass balance constant, c, which is selected to conserve the mass-balance of the catchment, the quick flow recession time constant, $\tau_{q}$, the slow flow recession time constant, $\tau_{s}$, and the proportion of effective rainfall which becomes slow-flow, $V_{s}$. For ephemeral streams [21]: the exponential loss parameter, *p*, to account for further loss of rainfall in the catchment and the soil moisture index threshold, *l*, a nonzero threshold value for rain to give streamflow.

The IHACRES model was incorporated into an open software environment for hydrological model assessment and development called ‘hydromad’ (Hydrological Model Assessment and Development) run in the R software system environment. The ‘hydromad’ package is focused on a top-down, spatially lumped, empirical approach to environmental hydrology [49].

In addition, the ‘hydromad’ package provides a set of functions that work together to construct, manipulate, analyze and compare hydrological models [50]. The class of hydrological models considered are dynamic (typically at a daily time step), spatially aggregated conceptual or statistical models [50]. The notable hydrological models incorporated into ‘hydromad’ include IHACRES catchment wetness index (CWI) model, IHACRES catchment moisture deficit (CMD) model, Sacramento soil moisture accounting model, *the modele du Genie Rural a 4 parametres Journalier* (GR4 J model), the Australian water balance model (AWBM), the single-bucket models of Bai et al. [51] and a degree-day factor snowmelt model of Kokkonen et al. [52].

The ‘hydromad’ package includes numerous varying routing models and several different optimization algorithms to simulate runoff from input data of rainfall, temperature/potential evapotranspiration and streamflow making it very efficient for this purpose. Details of the ‘hydromad’ package can be found in Andrews at al. [50]. The implementation of this study was done in R version 3.6.1 [53,54] using the formatR [55], zoo [56], latticeExtra [57], polynom [58], car [59], Hmisc [60], reshape [61], dream [62], coda [63], DEoptim [64], chron [65], hydromad [66] and sensitivity [67] packages with all their dependencies. The daily evapotranspiration data acquired were transformed by simple disaggregation into hourly evapotranspiration data (as done by Bennett et al. [26] with rainfall data) to use in this study since the temporal data resolution was in hours. We implemented the IHACRES CWI model for this study and the Nash–Sutcliffe Efficiency (R^2^ in IHACRES and ‘hydromad’) as the objective function.

The model implementation in RStudio [68] was initialized by importing the data (a time series in this case) into the program. This was done after the installation and loading of the required R packages necessary for the success of the model application in the R statistical software environment. The time series imported into R had to be in a usable format. This means the structure of the imported data should be recognized by R in the way the user intends, especially in the date format and data units for the purpose at hand. For example, when data considered as a time series by a user was imported into the program, R may recognize the data as a data frame with the date column as factors as witnessed in this study. Therefore, the user needs to transform the imported data into a time series in R to be able to be used for the intended purpose. Since the data used in this study were hourly, the date column in this study was transformed into an R recognizable format using the chron package with the POSIXct function and setting the time zone of the imported data. The zoo package was then used to transform the data into an R available time series and also set the time resolution frequency. This allows you to plot and visualize the imported data properly in R.

In addition, ‘hydromad’ model fitting functions require that rainfall and streamflow were given in the same units [49]. Therefore, the runoff in m^3^/s, was converted to mm/hour, the same units as the rainfall data in this study. The conversion of the runoff from m^3^/s to mm/hour was done by averaging the flow volume over the watershed area (given in km^2^); which must be applied when converting between measures of depth and measures of volume. The ‘hydromad’ model object to be applied for the modeling purpose was then specified.

At this stage, the Nash–Sutcliffe efficiency measure (NSE or E or R^2^) [36] calculated as:

(1) $$NSE\;or\;R^{2}=1-\frac{\sum_{i=1}^{n}{\left(O_{i}-P_{i}\right)}^{2}}{\sum_{i=1}^{n}{\left(O_{i}-\bar{O}\right)}^{2}}.$$

where O = observed values, P = predicted values, $\bar{O}$ = mean observed values and *n* = number of cases, with R^2^ ranging from −∞ to 1 and R^2^ > 0.50 considered acceptable [39], was used as the objective function in this study. This means that the R^2^ was used in the optimization procedure as the stopping criteria when the parameters sampled from the parameter response surface produced the highest efficiency value.

The preferred time aggregation of the objective function; that is the objective function at the daily scale (rsq_daily) or hourly scale (rsq_hourly), was then selected before the model was calibrated by optimization. The results were then plotted and visualized for interpretation.

The Morris global sensitivity analysis [69] was run after the model calibration to investigate and compare the parameter sensitivities when the rsq_daily and rsq_hourly objective functions were employed in the modeling process. The Morris global sensitivity analysis generates sensitivity indices in the form of the mean (μ) (absolute mean $\left(\mu^{*}\right)$ in ‘hydromad’ which resolves the problem of the effects of opposite signs due to a model’s non-monotonic characteristics suggested by Campolongo et al. [70]) and standard deviation (σ) values, which designate the influence of each parameter on the target function [71]. A high $\mu^{*}$ value suggest that a parameter has an important overall influence on the target function, and a high value of σ indicating that a parameter has strong interactions with other parameters (that is how strongly one parameter interacts with other parameters) or that the effect of the parameter is nonlinear [69,72].

The sensitivity analysis was performed on events that produced acceptable model performance results based on the goodness of fit measures obtained after model calibration, notwithstanding the objective function applied (objective function at a daily scale or hourly scale). This was done to reflect on the sensitivities of feasible calibrated parameters for the model as a whole regardless of the objective function scenarios. That is, the sensitivity analysis was performed to assess whether there were changes in the parameter sensitivities between the objective functions used. Details on the use of IHACRES in ‘hydromad’ in R can be obtained in Andrews [49] and the use of the Morris global sensitivity analysis in ‘hydromad’ in R in Shin et al. [71]. The default R^2^ in ‘hydromad’ used to assess model performance is the monthly R^2^, although other functions are also available. ‘hydromad’ also automatically provides the relative bias (i.e., bias as a fraction of the total observed flow), R^2^ square root (R-squared using square-root-transformed data) and R^2^ log (R-squared using log-transformed data, with an offset) measures.

Several different model combinations were run to obtain the best model structure to model the flood events in the Zhidan catchment. The simple, refined instrumental variable (SRIV) routing model (which allows the user to select the configuration of stores that best describes the catchment of interest) of a single exponential store (1, 0) was selected. The default monthly R^2^ in ‘hydromad’ could not be applied as the objective function for the data in this study because the event data periods were not up to a month for any of the events, as presented in the exploratory data analysis results of Table 1. Thus, the need to generate objective functions with different temporal aggregations for this study and in cases with data such as this. The temporal aggregation or time aggregation of the objective function is done to change the default time structure of the objective function, which is monthly in ‘hydromad’, into the preferred time of the user (hourly and daily in this study).

To execute the objective function constraints in this study, the Nash–Sutcliffe Efficiency (R^2^ in IHACRES) objective function applied in this study was diversified into daily efficiency measures (the efficiency measured at a daily scale (rsq_daily)) and hourly efficiency measures (the efficiency measured at an hourly scale (rsq_hourly)) for all the selected events to assess the implications of this objective function constraint on the performance of the model. ‘hydromad’ in R allows the user to generate objective functions with temporal aggregation with the buildTsObjective() function and this was used to build the daily and hourly R^2^ objective functions. More information about the time aggregation of objective functions in ‘hydromad’ can be found at http://hydromad.catchment.org/#buildTsObjective. The relative bias and root mean square error (RMSE) measures were also added to the model performance measures to evaluate the general performance of the IHACRES model, calculated as:

(2) $$Relative\;bias=\frac{\sum_{i=1}^{n}\left(P_{i}-O_{i}\right)}{\sum_{i=1}^{n}O_{i}}.$$

Root Mean Square Error (RMSE) [39,73]:

(3) $$\mathrm{RMSE}=\sqrt{\frac{1}{n}\sum_{i=1}^{n}{\left(O_{i}-P_{i}\right)}^{2}}.$$

where O = observed values, P = predicted values and *n* = number of cases.

Finally, in modeling event flows, an accurate prediction of peak flows also imperative to investigate the ability of the built models to suitably predict flood event runoff peaks and so the Percent Error in Peak Flow (PEPF) [74,75] calculated below was introduced as an additional model performance measure.

(4) $$PEPF=100\left|\frac{\left|\left(O_{P})-(P_{P}\right)\right|}{\left(O_{P}\right)}\right|.$$

where $O_{P}$ and $P_{P}$ are observed and predicted peak discharge, respectively. PEPF closer to zero means a better estimation of peak flow by the IHACRES model. The satisfactory value of PEPF in this study is considered as ≤25% of the difference in observed and simulated peak flows.

The parameter uncertainty associated with the results of the model simulation based on the different objective function choices, which in IHACRES is assessed with the percent average relative parameter error (% ARPE) measure, used to evaluate the correctness of the calibration [76] is also considered. The ARPE is an integral part of the simple, refined instrumental variables (SRIV) calibration method and is given by Jakeman et al. [17] as:

(5) $$ARPE\left(n,m\right)=\frac{1}{\left(m+n+1\right)}\left[\sum_{i=1}^{n}\frac{{\hat{\sigma }}_{i}^{2}}{{\hat{a}}_{i}^{2}}+\sum_{i=0}^{m}\frac{{\hat{\sigma }}_{i+n+1}^{2}}{{\hat{b}}_{i}^{2}}\right].$$

where m and n are the orders of the polynomials of the transfer function indicating the most appropriate model structure and parameterization, ${\hat{\sigma }}_{i}^{2}$ is the estimated parameter variance which is the estimated variance of the i-th element in $\hat{a}$ = ($a_{1},\;a_{2},\;\ldots ,\;a_{n},\;b_{0},\;b_{1},\;\ldots ,\;b_{m}{)}^{T}$, with the superscript T denoting the vector transpose. These variances are available as byproducts of the SRIV algorithm [17].

The code used for executing the modeling process in this study in R is provided in Appendix A.

## 3. Results

### 3.1. Exploratory Data Analysis Results

The period statistics of the selected flood events of the Zhidan watershed modeled in this study are presented in Table 1.

As observed from Table 1, the flood events with the longest data points are the years 2002b and 2005 with 61 h of data translating into about 2.5 days of data while the flood event with the least data points occurred in 2000, with 22 h of data and not making up to one (1) day. The variation occurring in the Zhidan catchment is presented in Table 1. Table 1 demonstrates that the short duration and somewhat low rainfall peak of the flood event in the year 2000 resulted in quite a significant runoff peak, showing the close proportionality of the rainfall–runoff relationship in the catchment in that year. This could be as a result of uninterrupted flow throughout the catchment to the catchment outlet and thus suggesting fewer human interventions in the watershed in that period.

In addition, the year 2002 is seen to be dominated by more events than the other years with some high rainfall and runoff peaks. This suggests the impacts of climate variability in the Zhidan watershed leading to a wet year. Furthermore, the rainfall and runoff peaks of the year 2003 are seen to be considerably low, suggesting more climate variability and the development of hydraulic infrastructures in the watershed, their effects being visible in succeeding years (2005 and 2006). This is the result of studies reporting 615 warping dams (soil-retaining dams for sediment control) with a total storage of 830 million m^3^ built-in Zhidan County, where the Zhidan catchment is located [41].

In addition, the changing catchment characteristics from the year 2000 to the year 2006 is evident in Table 1, where the runoff peaks in the latter years are observed to diminish substantially, regardless of the rainfall intensity or duration of the flood. For example, the runoff peak of the 2005 flood event is lower than that of the 2000 and 2002b flood events even though the rainfall peak of the 2005 flood event is greater than that for the events of 2000 and 2002b. This indicates the effects of significant land-use changes on the catchment properties in the latter event years.

**Figure 3:**
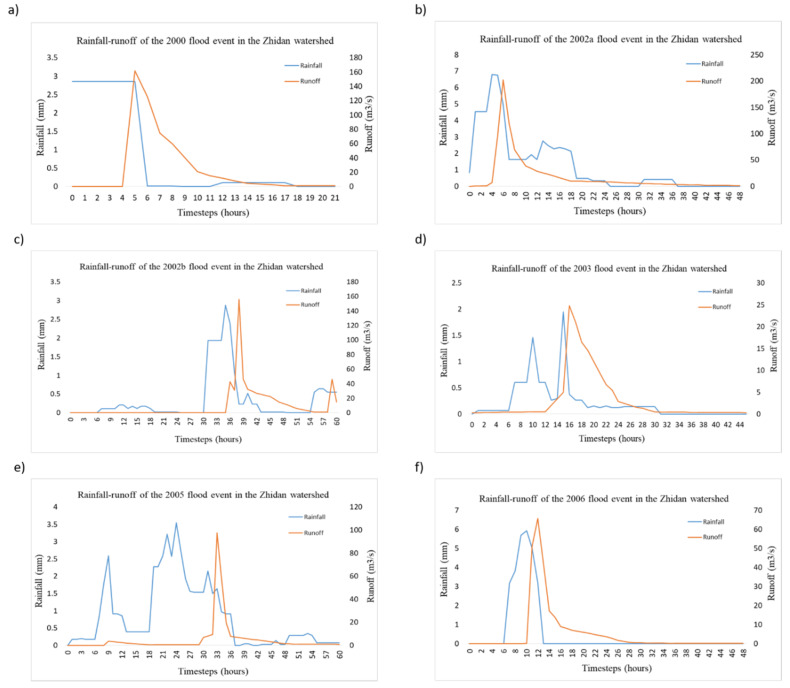
Rainfall–runoff relationships of the (**a**) 2000, (**b**) 2002a, (**c**) 2002b, (**d**) 2003, (**e**) 2005 and (**f**) 2006 flood events.

The results of the visual exploratory data analysis performed on the selected flood events of the Zhidan watershed are presented in Figure 3, which illustrates the rainfall–runoff relationship for each selected year with the blue and red lines signifying rainfall and runoff, respectively.

The results of the visual exploratory data analysis display the rainfall–runoff relationship in the Zhidan watershed to be extensively complex. For example, in the 2000 flood event (Figure 3a), the runoff peak is observed to be occurring immediately after the rainfall peak without much delay, meaning the somewhat high-intensity rainfall from the start of the event was quickly transferred to the catchment outlet to have this form of rainfall–runoff relationship. In addition, for the 2002a (Figure 3b) and 2002b (Figure 3c) flood events, the runoff peaks are witnessed to closely follow the rainfall peaks showing a short lag time between the rainfall and runoff in the watershed for these events due to less anthropogenic interventions.

However, for the 2003 (Figure 3d) and 2005 (Figure 3e) flood events, the runoff is observed to be unaffected by several rainfall peaks, having a single abrupt peak in the middle of the event, which then decreases abruptly towards the end of the event. This observation depicts that multiple rainfall peaks do not result in multiple runoff peaks, possibly due to interception storage in the Zhidan watershed caused by land-use changes in the watershed during these years following the numerous warping dams reported.

Finally, the rainfall–runoff relationship for the 2006 flood event (Figure 3f) returns to having a single rainfall peak and a single runoff peak with a short lag time between the rainfall peak and the runoff peak similar to the 2002a and 2002b flood events.

Looking at the rainfall–runoff relationship of the flood events of the Zhidan catchment, it can be concluded that meteorological variability affecting rainfall and land-use changes, affecting runoff, played extremely significant roles affecting floods in the region. Therefore, studies into these role players will aid in better understanding and management of flood risks and water resources in the watershed. These observations of the Zhidan watershed deepens the requirement of further study into the hydrological characteristics and changes in semi-arid regions since they seem to be majorly affected by the slightest changes in their environments.

### 3.2. Model Results

Table 2 displays the identification of unit hydrographs and component flows from rainfall, evapotranspiration and streamflow data (IHACRES) model performance statistics after simulating the different flood events with the rsq_daily and rsq_hourly objective function constraints. From Table 2, the large differences in the model performance of the different objective functions are observed, especially in the R^2^ measures. The average relative parameter error (ARPE) results are also witnessed to improve extensively from rsq_daily to rsq_hourly, especially for the 2002a, 2003, and 2005 flood events. Furthermore, the use of the rsq_hourly objective function saw an improvement in the simulation of the peak flows in all the studied events, however with most estimated peak flow values to be above the maximum acceptable value in this study except the 2003 and 2006 flood events using the rsq_hourly objective function.

The changes in the relative bias (rel. bias) and root mean square error (RMSE) measures are however minor, and they showed good performance for all modeled events irrespective of the objective function applied, although the RMSE for the rsq_hourly events is better.

**Figure 4:**
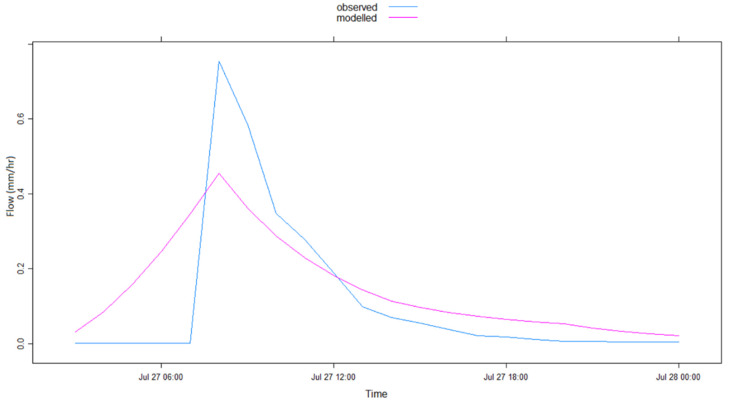
IHACRES model simulation results with rsq_hourly objective function for the 2000 flood event. IHACRES—identification of unit hydrographs and component flows from rainfall, evapotranspiration and streamflow data.

**Figure 5:**
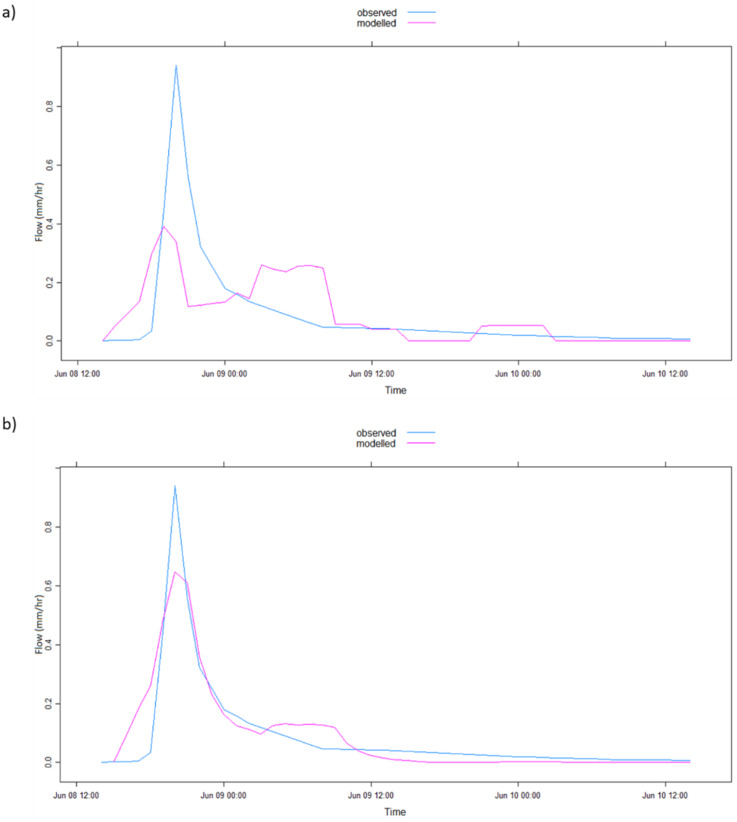
IHACRES model simulation results with (**a**) rsq_daily and (**b**) rsq_hourly objective functions for the 2002a flood event.

**Figure 6:**
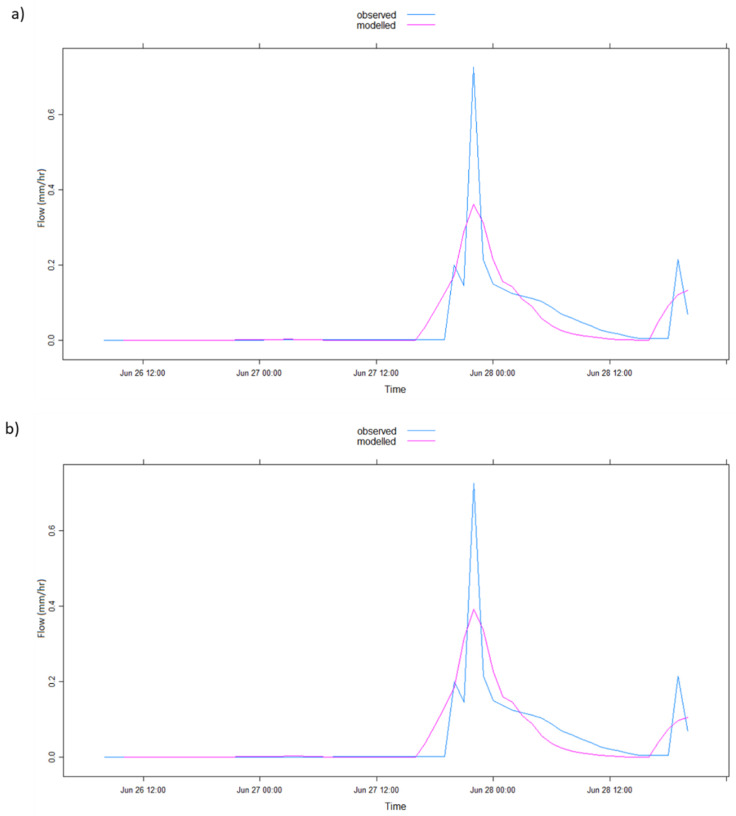
IHACRES model simulation results with (**a**) rsq_daily and (**b**) rsq_hourly objective functions for the 2002b flood event.

**Figure 7:**
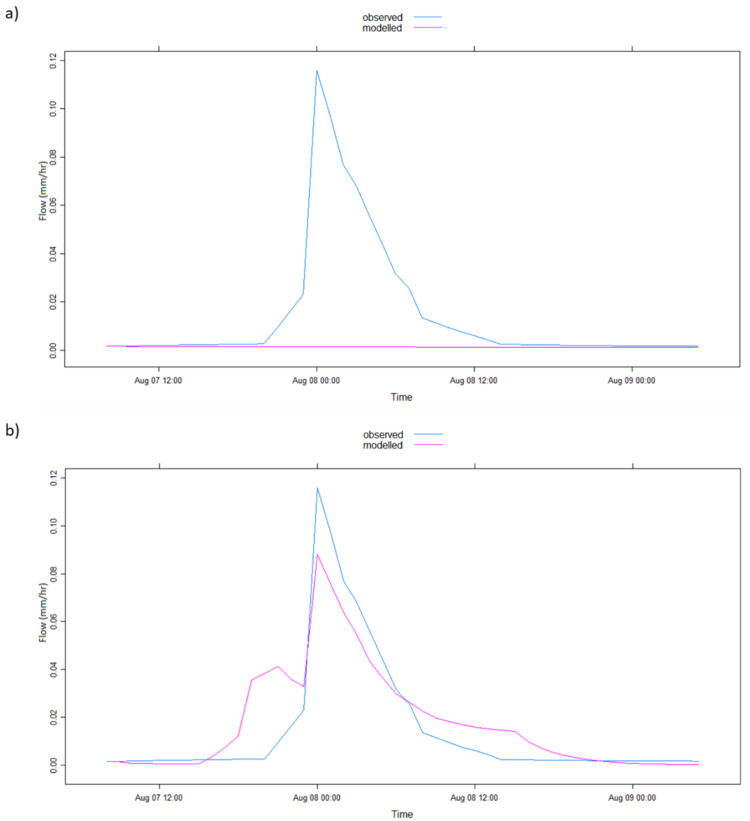
IHACRES model simulation results with (**a**) rsq_daily and (**b**) rsq_hourly objective functions for the 2003 flood event.

**Figure 8:**
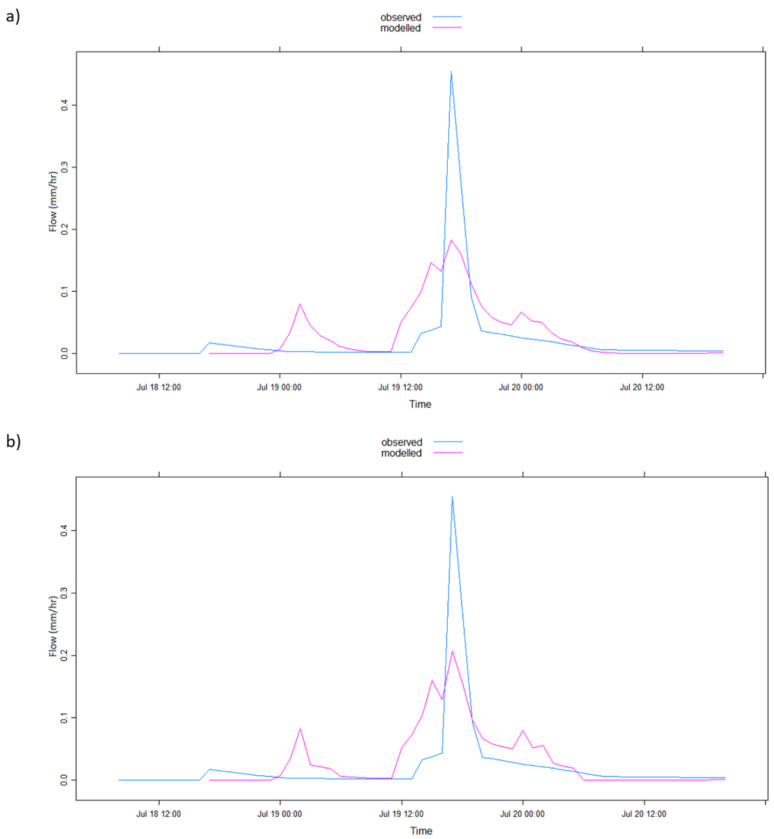
IHACRES model simulation results with (**a**) rsq_daily and (**b**) rsq_hourly objective functions for the 2005 flood event.

**Figure 9:**
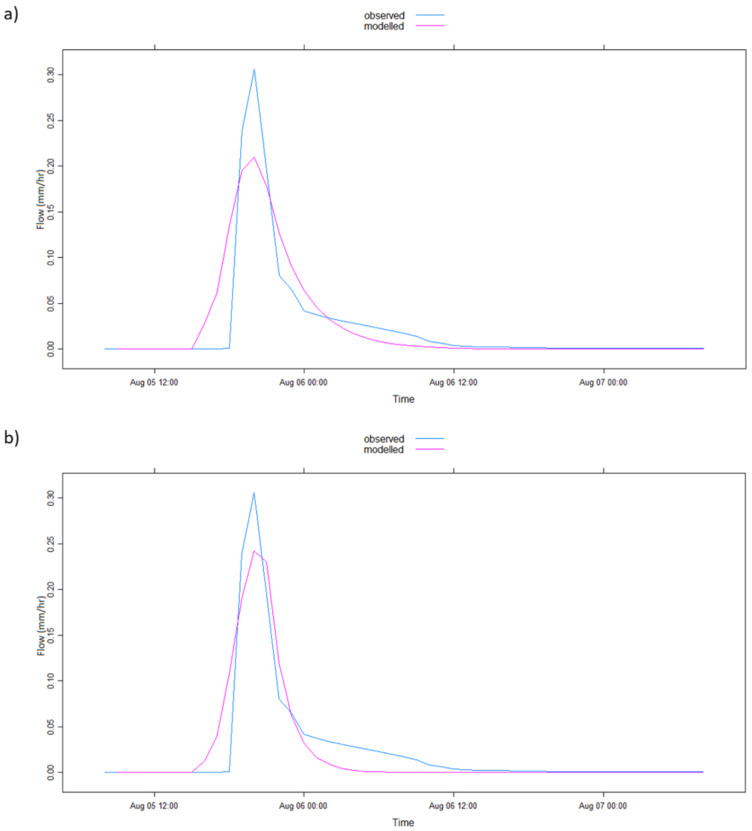
IHACRES model simulation results with (**a**) rsq_daily and (**b**) rsq_hourly objective functions for the 2006 flood events.

The graphical representation of the model simulation results in Table 2 is illustrated in Figure 4, Figure 5, Figure 6, Figure 7, Figure 8 and Figure 9 where Figure 5a, Figure 6a, Figure 7a, Figure 8a, Figure 9a represent observed and simulated hydrographs using the rsq_daily objective function and Figure 4, Figure 5b, Figure 6b, Figure 7b, Figure 8b and Figure 9b display the observed and simulated hydrographs using the rsq_hourly measure, with the blue and red lines showing the observed and modeled hydrographs, respectively.

The application of the rsq_daily objective function to the flood event of the year 2000 resulted in non-convergence in the optimization of the R^2^ objective function because the data points of that particular flood were not more than 1 day (24 h). Therefore, the rsq_daily objective function failed (Table 2). Figure 4, therefore, shows the results of modeling the 2000 flood event with the rsq_hourly objective function. It can be witnessed from Table 2 and Figure 4 that the application of the rsq_hourly objective function to the flood event of the year 2000 produced satisfactory model simulation results, thus showing the efficacy of the rsq_hourly objective function in limited data situations.

In addition, Figure 5 exhibits the observed and simulated hydrographs for the 2002a flood event. Figure 5b demonstrates the tremendous enhancement in model performance from Figure 5a in terms of the overall hydrograph shape from the beginning to the end of the event and the better-estimated peak flow by the application of the rsq_hourly objective function in Figure 5b. This overall improvement of model performance is also observed in differences in R^2^, Percent Error in Peak Flow (PEPF) and ARPE values in Table 2 for the 2002a flood event.

The ARPE for the 2002a flood event when the rsq_daily objective function was applied is highly abnormal, which shows the difficulty of the IHACRES model to obtain feasible parameters for that particular event in the application of the rsq_daily objective function. This challenge is rectified by the use of the rsq_hourly objective function, as observed in Table 2. The differences in model performance of the 2002b flood event are subtle in the hydrograph comparison graph of Figure 6. However, a closer look at Figure 6b shows a peakier and enhanced simulated peak using the rsq_hourly objective function leading to slightly enhanced R^2^ and PEPF values than when the rsq_daily objective function was applied (Table 2).

Furthermore, the exceptional turnaround of the model performance in the 2003 flood event between the objective functions is witnessed in Figure 7. Figure 7a showed extremely poor model performance when the rsq_daily objective function was employed in the simulation of the 2003 flood event; the simulated hydrograph seen as a straight line. This is presented clearly in Table 2, where the R^2^ is −0.26, the PEPF is 98.65%, and the ARPE—the parameter error measure—is immeasurable, portraying the worse model performance throughout the entire study.

However, Figure 7b shows a whole different model performance status when the rsq_hourly measure was adopted where the simulated hydrograph follows the shape of the observed hydrograph and the simulated peak observed to be close to the observed peak. This is witnessed in Table 2, where the R^2^ is 0.79, the PEPF is 23.97%, well within the acceptable range, and the ARPE is a minor value of 0.15%. This upgrade of model performance in the 2003 flood event shows the advantage of the application of the rsq_hourly objective function to make use of the fundamental time steps of the data.

Similar to the 2002b flood event (Figure 6), the differences in the model performance between the two objective functions in the 2005 flood event illustrated in Figure 8 are faintly detected, except the boost in the simulated peak flow of Figure 8b, meliorating the model performance using the rsq_hourly measure. This slight enhancement in model performance is also observed in Table 2 where all the improvements are small (the R^2^ upgrading from a poor simulated value of 0.49 with rsq_daily to a satisfactory value of 0.52 according to Moriasi et al. [39] using rsq_hourly). However, the ARPE value improves from 5.53% in the use of the rsq_daily objective function to 0.01% when the rsq_hourly measure was applied, considered significant in this study because of the volatile nature of parameter changes.

Finally, Figure 9 represents the observed and modeled event runoff of the 2006 flood event. Figure 9b shows a better estimation of the peak flow than Figure 9a, while all other estimations remain approximately the same. Because of this upgraded peak flow simulation in Figure 9b, an improvement in R^2^ and PEPF is recorded in the model performance with rsq_hourly objective function in Table 2. However, it can be witnessed from Table 2 that the ARPE value increased from 0.14% in the application of rsq_daily to 0.43% in using rsq_hourly, although marginally. This depreciation in ARPE values from rsq_daily to rsq_hourly could represent a greater non-uniqueness of the rsq_hourly parameters than their rsq_daily counterpart.

Using the stated consideration for the sensitivity analysis, the events occurring in 2002b and 2006 were the only flood events that produced acceptable R^2^ model performance values for both objective function scenarios, as witnessed in Table 2. The calibrated parameters for the selected events are displayed in Table 3 to clarify the results of the sensitivity analysis and Table 4 illustrates the sensitivities of the parameters obtained for the rsq_daily and rsq_hourly objective functions during the 2002b and 2006 flood events. 

The sensitivity analysis results of the 2002b calibration flood event in the Zhidan catchment in Table 4 reveals the scale parameter as the most sensitive parameter with the highest $\mu^{*}$ and σ values (highly influencing the objective function results and having substantial interaction with all other parameters) followed by the v_s parameter whose interaction with the other parameters is more than its influence on the objective function, both being significantly high. The tw and tau_s parameters seem to have medium sensitivities, both having higher σ values than $\mu^{*}$ values, and the f parameter appears to be the least sensitive parameter influencing the model simulation results of the 2002b flood event in the Zhidan watershed. This observation is seen to be unchanging for both time aggregated objective function scenarios for the 2002b flood event. That is, the parameter sensitivities of the rsq_daily objective function are the same for the rsq_hourly objective function.

Table 4 also demonstrates the scale parameter as the most sensitive parameter for the 2006 event in the Zhidan catchment with the highest $\mu^{*}$ and σ values, with its interaction with the other parameters higher than its influence on the objective function. This is followed by the v_s parameter, also having a stronger interaction with the other parameters than influences the objective function. The tw, f and tau_s parameters appear to be the lesser sensitive parameters influencing the model simulation results, with the f parameter being least sensitive. The parameter sensitivities are also witnessed to be similar for both time aggregated objective functions for the 2006 flood event as for the 2002b flood event. The parameter sensitivity analysis results in Table 4 shows that the interaction between parameters has a stronger influence in the model process than their influence on the objective function for both selected flood events. This depicts the high interdependence of the model parameters on each other in simulating the streamflow events in the Zhidan watershed.

## 4. Discussion

The reason for the results obtained above is because: in the application of the rsq_daily objective function, the optimization algorithm searches the parameter space for optimal parameters which maximize R^2^ for each day represented in the data, whereas the rsq_hourly objective function seeks to maximize the R^2^ value for each hour represented in the data. Therefore, the use of rsq_hourly in this study resulted in consideration of the R^2^ value at every hour to be maximized rather than maximizing the R^2^ value for entire days in the case of rsq_daily and thus produced parameters accordingly for this purpose.

In this case, although the data are limited for the flood events, the rsq_hourly searches the parameter space considering ‘more data’ than its rsq_daily counterpart. For example, in this particular study, the flood events with the most data points have 61 h of data translating into about 2.5 days. Therefore, the rsq_hourly objective function searches the parameter space for optimal parameters to maximize R^2^ for 61 h. In contrast, the rsq_daily objective function generates parameters to maximize R^2^ for about 2.5 days. Likewise, in the 2000 flood event where the data period of the flood was less than one day, the application of the rsq_hourly objective function did not only result in convergence but obtained an R^2^ value of 0.57, considered satisfactory by Moriasi et al. [39] while the rsq_daily objective function failed.

This consideration of ‘more data’ by the rsq_hourly objective function is somewhat comparable to work by Bennett et al. [26], where model performance was better when calibrated with hourly data disaggregated from daily data because they gave the model more information. Moreover, since in data-driven modeling, data adequacy is one of the most crucial conditions influencing model performance, the rsq_hourly objective function has an advantage. Finally, the rsq_hourly objective function is especially useful when the available data are so scarce. It is not up to 24 h or more, which makes a typical day and thus making the rsq_daily measure inapplicable.

The main enhancement in model performance using the rsq_hourly objective function is observed to be the superior simulation of the high flows, which is the peak period for all the events. This enhancement is also because of the ‘more data’ concept, which appears to resolve the deficiency in peak flow simulation in the application of the rsq_daily objective function. This improvement in peak flow simulation is the reason behind the upgrade in R^2^ value from the rsq_daily to the rsq_hourly objective function because R^2^ is highly sensitive to high flows, and low flows tend to be neglected [38]. Nevertheless, all the simulated peaks for all the studied events, despite the objective function used, are underestimated and could be a weakness in the identification of unit hydrographs and component flows from rainfall, evapotranspiration and streamflow data (IHACRES) model for modeling flood events since its applications have been mostly for continuous modeling.

The average relative parameter error (ARPE) results which represent the parameter error and uncertainty obtained during the modeling of the flood events showed that the parameters obtained for the 2002a and 2003 flood events using the rsq_daily objective function may be exceptionally volatile resulting in significant errors in the parameters estimated or in the case of the 2003 flood event, this error could not be quantified. This could be as a result of the challenge of the IHACRES model to better quantify the considerable catchment changes during these flood events when the rsq_daily objective function is employed.

As can be witnessed in Table 1, the 2002a flood event possessed the highest rainfall and runoff peaks. In contrast, the 2003 flood event had the lowest rainfall and runoff peaks of all the studied events showing crucial changes in both meteorological variability and anthropogenic activities in the Zhidan catchment in those event years. The implementation of the rsq_hourly objective function is witnessed to aid the IHACRES model in this light with exceedingly satisfactory ARPE results for the 2002a and 2003 flood events (Table 2).

It should be noted that the main objectives for this study were the implementation and investigation of model performance using different time aggregations of the Nash–Sutcliffe objective function in a data-driven model for flood event modeling in a semi-arid watershed with data limitation issues to obtain enhanced model performance and feasible parameters for catchments when the default or commonly used model simulation processes fail due to data limitations. This is especially true in regards to semi-arid regions, which are mostly data-scarce, and studies focusing on the methodology in this work are lacking in hydrology.

However, to associate this study to time resolution dependency of parameters of studies as Littlewood and Croke [24], Reynolds et al. [25], Bennett et al. [26], Littlewood et al. [27], Littlewood and Croke [28] and Finnerty et al. [77] among others, a longer time series (both daily and sub-daily) from the Zhidan watershed would be required to test the hypothesis on this methodology to investigate whether parameters obtained from time aggregation of objective functions are independent of the data time step. That is, seeking to answer the question: can parameters obtained by employing time aggregations to objective functions be transferred successfully to data with the same temporal resolutions? For example, applying parameters acquired from implementing daily time aggregated objective functions to data of daily temporal resolution or parameters obtained with hourly time aggregated objective functions used on data with hourly temporal resolution. This would be the scope of future studies.

Taking into account the parameter sensitivity results, it is generally observed that although the different studied flood events using the different time aggregated R^2^ objective functions produced different parameters (Table 3), the parameter sensitivities remain the same for the different objective functions applied for each event. The sensitivity analysis results demonstrated the scale parameter as the most sensitive parameter followed by the v_s parameter, and the f parameter possessing the least sensitivity for all the considered events. This shows that both time aggregated objective functions had a good correlation as to the parameters. Therefore, there arises minimum or no uncertainty in parameter sensitivity changes across objective functions when the time aggregated R^2^ objective function is applied in later studies proving that the parameters continuously represent the catchment processes regardless of the objective function employed.

The work from this study has shown that it can be a challenging task to model streamflow in catchments with data limitations, especially in semi-arid regions, which mostly have rapid streamflow responses. However, the application of time aggregations to the objective functions selected during the calibration process could resolve this issue by obtaining better model performances and feasible parameters for use in these catchments without the requirements of additional data.

In this study, it has also been made evident that for a semi-arid region with much data limitations such as the Zhidan watershed where physical models have failed to simulate flood events better, the IHACRES model has proven successful and has satisfactorily modeled some flood events even with the rsq_daily objective function. This has proven the feasibility and capability of data-driven models as useful and efficient tools in modeling flood events in data-scarce semi-arid regions.

## 5. Conclusions

The comprehensive hydrological data required by conceptual or physically based models are rarely available in most watersheds and has led to the rapid development of data-driven models (DDMs) into hydrology and environmental sciences, which have demonstrated to be useful tools. In addition, the success of a calibration process is tremendously dependent on the objective function chosen as a calibration criterion and objective function constraints were applied in modeling to enhance variable simulations by models.

This study, therefore, applies the identification of unit hydrographs and component flows from rainfall, evapotranspiration and streamflow data (IHACRES) model, a hybrid conceptual-data-driven rainfall–runoff model to simulate a series of flood events with limited data over some years representing changes in land-use and landcover settings in Zhidan, a semi-arid catchment in northern China, applying objective function constraints in the form of diversifying the Nash–Sutcliffe efficiency (R^2^ in IHACRES) into daily (rsq_daily) and hourly (rsq_hourly) scales during the modeling process to investigate (1) the influence of objective function constraints on the model performance and (2) the general capability of the IHACRES model to simulate flood events in a data-scarce semi-arid region. The following conclusions were reached from the obtained results:

1. The model simulation results with the application of the rsq_hourly objective function were extensively better than the model performance with rsq_daily objective function because the rsq_hourly objective function seeks to maximize the R^2^ value for each hour represented in the data (which in this case is hourly flood data with limited data points). In contrast, the rsq_daily objective function maximizes R^2^ for each day represented in the data producing parameters accordingly. This provides the rsq_hourly measure an advantage over the rsq_daily objective function, such that in the present study, the optimization process with the utilization of the rsq_hourly measure considers ‘more data’ than its rsq_daily counterpart;
2. The IHACRES model struggled to satisfactorily simulate the flood peaks with a somewhat more accurate estimation of the flood at the start and end of each event, notwithstanding the objective function employed and thus could be considered a general model weakness in this study;
3. The IHACRES model implementation performed in simulating the selected flood events demonstrated the overall capability of the model to satisfactorily simulate flood events under changing land-use situations in a data-scarce semi-arid region with highly accurate parameters based on the parameter uncertainty results. This, therefore, endorses the use of data-driven models as hydrological models in data-scarce regions where physical models are inapplicable;
4. Finally, it is recommended that possible changes in time aggregation of objective functions be incorporated into models to account for modeling flood events with limited data like the Zhidan study area to make flood simulations in regions like this a possibility;
5. Further research could also be conducted with flood event data from other regions with data limitation constraints using time aggregated objective functions to compare the estimated R^2^ and parameter results; performing these comparisons across different climatic settings to investigate the behavior of objective function aggregations on data with different hydrological characteristics, however with more emphasis on semi-arid regions.

## Figures and Tables

**Table 1:** Observed statistics of the periods of the selected flood events of the Zhidan watershed.

Year	Start Time	End Time	Number of Event Days (days) *	Number of Data Points and Time Steps (hours)	Rainfall Peak (Rpeak) (mm)	Flow Peak (Qpeak) (m^3^/s)
2000	27 July 2000 3:00:00 a.m.	28 July 2000 12:00:00 a.m.	0.9167	22 (0–21)	2.8586	162
2002a	8 June 2002 2:00:00 PM	10 June 2002 2:00:00 PM	2.0417	49 (0–48)	6.7914	202
2002b	26 June 2002 8:00:00 a.m.	28 June 2002 8:00:00 PM	2.5417	61 (0–60)	2.8786	156
2003	7 August 2003 8:00:00 a.m.	9 August 2003 5:00:00 a.m.	1.9167	46 (0–45)	1.9529	24.89
2005	18 July 2005 8:00:00 a.m.	20 July 2005 8:00:00 PM	2.5417	61 (0–60)	3.5429	97.59
2006	5 August 2006 8:00:00 a.m.	7 August 2006 8:00:00 a.m.	2.0417	49 (0–48)	5.9486	65.80

* Number of event days were calculated using the number of data points.

**Table 2:** IHACRES model performance statistics with objective function constraints.

Year	Rsq_Daily Objective Function					Rsq_Hourly Objective Function				
	R^2^	Rel. Bias	RMSE (mm/hour)	PEPF (%)	ARPE (%)	R^2^	Rel. Bias	RMSE (mm/hour)	PEPF (%)	ARPE (%)
2000	–	–	–	–	–	0.5728	0.2780	0.1307	39.5621	0.2740
2002a	0.3249	−0.0036	0.1356	58.3754	94,207	0.8366	0.0168	0.0672	30.9741	1.087
2002b	0.6643	−0.0252	0.0618	50.0675	1.7280	0.6705	−0.0119	0.0612	45.8568	0.5453
2003	−0.2561	−0.9102	0.0295	98.6450	NAN *	0.7873	0.1925	0.0122	23.9683	0.1531
2005	0.4860	0.2313	0.0511	59.6843	5.5330	0.5215	0.2116	0.0493	54.3359	0.0101
2006	0.7835	0.0349	0.0283	31.4869	0.1352	0.8491	−0.1115	0.0236	20.8359	0.4330

* NAN = Not A Number, meaning the number is immeasurable. IHACRES—identification of unit hydrographs and component flows from rainfall, evapotranspiration and streamflow data.

**Table 3:** Calibrated parameters for the 2002b and 2006 flood events with different time aggregated objective functions in the Zhidan watershed.

Calibrated Parameters										
Year	2002b					2006				
	tw	f	scale	tau_s	v_s	tw	f	scale	tau_s	v_s
Rsq_daily	30	4	0.0137	2.1335	1	0	1.3146	0.3587	2.9339	0.0271
Rsq_hourly	22.7258	0.7214	0.1323	2.0703	0.1196	28.7393	3.9612	0.1774	1.5291	0.0142

where: tw = Catchment drying rate at a reference temperature, $\tau_{w}$; f = Temperature modulation parameter, f; scale = A scale factor estimated by mass balance with observed streamflow; tau_s = Slow flow recession time constant, $\tau_{s}$; v_s = Proportion of effective rainfall which becomes slow-flow, $V_{s}$

**Table 4:** Parameter sensitivities of the rsq_daily and rsq_hourly objective functions for the 2002b and 2006 flood events.

	2002b				2006			
	Rsq_Daily		Rsq_Hourly		Rsq_Daily		Rsq_Hourly	
	$\mu^{*}$	σ	$\mu^{*}$	σ	$\mu^{*}$	σ	$\mu^{*}$	σ
tw	0.2837	0.8055	0.2837	0.8055	0.0164	0.1268	0.0164	0.1268
f	0.0622	0.1547	0.0622	0.1547	0.0011	0.0040	0.0011	0.0040
scale	0.7913	1.2566	0.7913	1.2566	0.6120	1.3991	0.6120	1.3991
tau_s	0.2304	0.8443	0.2304	0.8443	0.0428	0.1492	0.0428	0.1492
v_s	0.6632	1.1002	0.6632	1.1002	0.1598	0.5262	0.1598	0.5262

## Appendix A

R code used in this study.

install.packages(c(“formatR”, “zoo”, “latticeExtra”, “polynom”, “car”, “Hmisc”,”reshape”))

install.packages(“hydromad”, repos = “http://hydromad.catchment.org”)

install.packages(c(“DEoptim”, “coda”))

install.packages(“dream”, repos = “http://hydromad.catchment.org”)

install.packages(“chron”)

install.packages(“sensitivity”)

## Comment the above after installation

library(zoo)

library(dream)

library(coda)

library(DEoptim)

library(hydromad)

library(chron)

library(sensitivity)

#setting my working directory

setwd(“D:/RESEARCH/All things R/IHACRES”)

# Load in data

mdwhydromadtrialraw <- read.csv(file = file.choose(), header = F)

View(mdwhydromadtrialraw)

names(mdwhydromadtrialraw) <- c(“Date”, “P”, “Q”, “E”)

View(mdwhydromadtrialraw)

mdwhydromadtrial <- mdwhydromadtrialraw

# Chron can only use character or numeric dates, but R reads my dates as factors. So we convert to characters

datetimes = as.character(mdwhydromadtrial$Date)

# Create zoo object

chron_dt = as.chron(as.POSIXct(datetimes, format = “%d-%m-%Y %H:%M”, tz = “UTC”))

mdw_zoo = zoo(mdwhydromadtrial[, 2:4], order.by = chron_dt)

View(mdw_zoo)

# in hydromad the flow units must be the same as the rainfall units

# converting the flow

mdw_zoo$Q = convertFlow(mdw_zoo$Q, from = “cumecs”, to = “mm / hour”, area.km2 = 774)

mdwtrialts = zoo(mdw_zoo, frequency = 24)

summary(mdwtrialts)

# ploting the data to make sure everything worked

xyplot(mdwtrialts)

# Set seed value for reproducible results

set.seed(21)

# Using hydromad

# set warmup this way

hydromad.options(warmup = 0)

# The IHACRES CWI model in ‘hydromad’ was built this way

mdwtrialMod = hydromad(mdw_zoo,

      sma = “cwi”,

      routing = “expuh”, rfit = list(“sriv”, order = c(n = 1,m = 0)),

      tw = c(0,30),

      f = c(0,4),

      tau_s = c(0, 500),

      scale = c(0,0.5),

      v_s = c(0, 1))

# Changing the objective function time structure to daily instead of the default monthly. Use this function in the application of rsq_daily as objective function. Comment if not in use

rsq_daily <- function(Q, X, …) {

  .(buildTsObjective(Q, groups = cut(time(Q), “days”)))(Q, X, …)

}

# Changing the objective function time structure to hourly instead of the default monthly. Use this function in the application of rsq_hourly as objective function. Comment if not in use

rsq_hourly <- function(Q, X, …) {

  .(buildTsObjective(Q, groups = cut(as.POSIXct(time(Q)), breaks = “hour”)))(Q, X, …)

}

# Calibration using rsq_daily. Comment if not in use

mdwtrialFit = fitByOptim(mdwtrialMod, objective = rsq_daily, method = “PORT”, samples = 500)

# Calibration with rsq_hourly. Comment if not in use

mdwtrialFit = fitByOptim(mdwtrialMod, objective = rsq_hourly, method = “PORT”, samples = 500)

xyplot(mdwtrialFit)

summary(mdwtrialFit)  #hydromad automatically generates the R^2^ and rel. bias values at this step

summary(mdwtrialFit,stats = c(“ARPE”,”RMSE”))

fit = fitted(mdwtrialFit)  #To obtain simulated runoff for PEPF calculation

bfit = as.data.frame(fit)

View(bfit)

OQ = (mdw_zoo$Q)   #To obtain converted observed runoff for PEPF calculation

View(OQ)

## Run Morris Sensitivity analysis

mm <- morris(

  ## Utility function to evaluate parameters on a model

  model = evalPars,

  ## Names of factors/parameters

  factors = names(getFreeParsRanges(mdwtrialMod)),

  ## Number of repetitions

  r = 100,

  ## List specifying design type and its parameters

  design = list(type = “oat”,levels = 10,grid.jump = 2),

  ## Minimum value of each non-fixed parameter

  binf = sapply(getFreeParsRanges(mdwtrialMod), min),

  ## Maximum value of each non-fixed parameter

  bsup = sapply(getFreeParsRanges(mdwtrialMod), max),

  ## Hydromad model object to use to evaluate parameters

  object = mdwtrialFit,

  ## NSE* objective function

  objective = ~ hmadstat(“r.squared”)(Q, X) /

  (2-hmadstat(“r.squared”)(Q, X))

)

print(mm)
